# Human cytomegalovirus protein RL1 degrades the antiviral factor SLFN11 via recruitment of the CRL4 E3 ubiquitin ligase complex

**DOI:** 10.1073/pnas.2108173119

**Published:** 2022-02-01

**Authors:** Katie Nightingale, Martin Potts, Leah M. Hunter, Ceri A. Fielding, Cassie M. Zerbe, Alice Fletcher-Etherington, Luis Nobre, Eddie C. Y. Wang, Blair L. Strang, Jack W. Houghton, Robin Antrobus, Nicolas M. Suarez, Jenna Nichols, Andrew J. Davison, Richard J. Stanton, Michael P. Weekes

**Affiliations:** ^a^Cambridge Institute for Medical Research, University of Cambridge, Cambridge CB2 0XY, United Kingdom;; ^b^Department of Medicine, University of Cambridge, Cambridge CB2 0XY, United Kingdom;; ^c^Division of Infection and Immunity, Cardiff University School of Medicine, Cardiff CF14 4XN, United Kingdom;; ^d^Institute for Infection and Immunity, University of London, London SW17 0RE, United Kingdom;; ^e^Centre for Virus Research, Medical Research Council, University of Glasgow, Glasgow G61 1QH, United Kingdom

**Keywords:** human cytomegalovirus, restriction factor, innate immunity, host–pathogen interaction, Schlafen

## Abstract

Human cytomegalovirus (HCMV) is an important human pathogen and a paradigm of viral immune evasion, targeting intrinsic, innate, and adaptive immunity. We have employed two orthogonal multiplexed tandem mass tag-based proteomic screens to identify host proteins down-regulated by viral factors expressed during the latest phases of viral infection. This approach revealed that the HIV-1 restriction factor Schlafen-11 (SLFN11) was degraded by the poorly characterized, late-expressed HCMV protein RL1, via recruitment of the Cullin4-RING E3 Ubiquitin Ligase (CRL4) complex. SLFN11 potently restricted HCMV infection, inhibiting the formation and spread of viral plaques. Overall, we show that a restriction factor previously thought only to inhibit RNA viruses additionally restricts HCMV. We define the mechanism of viral antagonism and also describe an important resource for revealing additional molecules of importance in antiviral innate immunity and viral immune evasion.

## Significance

Previous proteomic analyses of host factors targeted for down-regulation by human cytomegalovirus (HCMV) have focused on early or intermediate stages of infection. Using multiplexed proteomics, we have systematically identified viral factors that target each host protein down-regulated during the latest stage of infection, after the onset of viral DNA replication. Schlafen-11 (SLFN11), an interferon-stimulated gene and restriction factor for retroviruses and certain RNA viruses, potently restricted HCMV infection. Our discovery that the late-expressed HCMV protein RL1 targets SLFN11 for proteasomal degradation provides evidence for a viral antagonist of this critical cellular protein. We therefore redefine SLFN11 as an important factor that targets DNA viruses as well as RNA viruses, offering therapeutic potential via molecules that inhibit RL1-mediated SLFN11 degradation.

Human cytomegalovirus (HCMV) is a ubiquitous pathogen that establishes a lifelong latent infection in the majority of the world’s population (1). Reactivation from latency in immunocompromised individuals, such as transplant recipients and AIDS patients, can result in significant morbidity and mortality (2). HCMV is also the leading cause of infectious congenital birth defects, including deafness and intellectual disability, affecting ∼1 in 100 pregnancies (1). However, only a few antiviral drugs are approved for the treatment of HCMV, all of which are associated with significant toxicity, and there is currently no licensed vaccine (3).

Susceptibility to viral infection and disease is determined in part by antiviral restriction factors (ARFs) and the viral antagonists that have evolved to degrade them (4). Small molecules that inhibit ARF-antagonist interactions may restore endogenous restriction and offer novel therapeutic potential (5). Identification of novel ARFs and characterization of their interactions with HCMV antagonists is therefore clinically important.

HCMV possesses the largest human herpesvirus genome, encoding 170 canonical open reading frames (ORFs). A modest number of noncanonical ORFs may encode additional functional proteins (6–9). During productive HCMV infection, viral gene expression occurs in cascades during an ∼96-h infection cycle that is conventionally divided into immediate-early, early, and late phases. Early genes encode functions necessary for initiating viral DNA replication. In the late phase, early-late genes are initially transcribed at low levels and are then up-regulated after the onset of viral DNA replication, whereas true-late genes are expressed exclusively after DNA replication commences and include proteins required for HCMV virion assembly. We previously characterized five temporal classes of viral protein expression, offering finer definition of protein expression profiles (10).

As over 900 proteins are down-regulated more than threefold during the course of HCMV infection, predicting molecules likely to perform novel immune functions is challenging without additional data (7, 10, 11). Our previous analysis of the subset of proteins targeted for degradation by 24 or 48 h led directly to the identification of helicase-like transcription factor (HLTF) as a novel ARF, and HCMV UL36 as a key inhibitor of necroptosis, by degrading mixed-lineage kinase domain-like protein (7, 10). However, no studies have systematically examined which host factors are targeted by viral proteins during the latest phase of infection. This question is important as some host factors may play important roles in restricting the final stages of viral replication. Furthermore, despite our prior characterization of a comprehensive HCMV interactome (9), the abundance of certain host proteins whose expression is down-regulated during infection can be sufficiently low to impede identification of their viral antagonists.

We have used two complementary proteomic approaches to address these questions. The first identified cellular proteins specifically targeted by HCMV factors expressed after viral DNA replication, by comparing host protein expression over time in the presence or absence of the viral DNA polymerase inhibitor phosphonoformic acid (PFA). The second employed an enhanced panel of HCMV mutants each deleted in contiguous gene blocks dispensable for virus replication in vitro, most of which we have described previously (12).

The intersection between these approaches showed that one particular protein, Schlafen family member 11 (SLFN11), is both down-regulated during the late phase of HCMV infection and is targeted by the RL1-6 block of viral genes. SLFN11 potently restricted HCMV infection and therefore represents a unique HCMV ARF. Among the factors encoded by the RL1-6 region, RL1 was required for SLFN11 down-regulation, via recruitment of the Cullin4-RING E3 Ubiquitin Ligase (CRL4) complex. Overall, our data identify a HCMV ARF and a unique mechanism of viral antagonism, and describes an important resource that will reveal additional molecules of importance in antiviral innate immunity and viral immune evasion.

## Results

### Host Proteins Down-Regulated by Late-Expressed HCMV Factors.

**Fig. 1.**
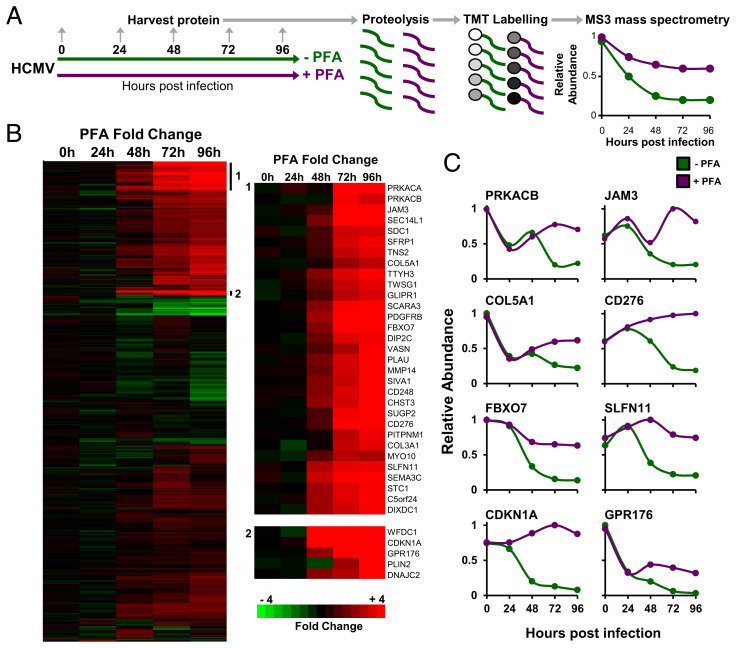
Host proteins targeted for down-regulation by HCMV late during infection, identified using the viral DNA synthesis inhibitor PFA. (*A*) Schematic of the experimental workflow. HFFFs were infected with HCMV at an MOI of 10, and cells were harvested at the indicated times. A high MOI was chosen in order to be consistent with our previous publications (7, 10, 11), and to infect as close as possible 100% of cells. This ensured that the ratios for protein down-regulation were not compressed by proteins expressed (and not down-regulated) in uninfected cells. (*B*) Hierarchical cluster analysis of 527 proteins down-regulated ≥3-fold by 96 hpi. For each protein, the ratios of protein expression in the presence or absence of PFA are shown. To be considered a “hit” in the screen, proteins were additionally required to be rescued >2-fold by PFA. Enlargements to the right of the panel show examples of subclusters. (*C*) Examples of temporal profiles of proteins rescued from down-regulation by PFA.

To globally quantify cellular proteins whose expression is increased or decreased by late-expressed HCMV factors, we applied PFA to HCMV-infected primary human fetal foreskin fibroblasts (HFFFs) at the time of infection and harvested samples for analysis at 24-h intervals (Fig. 1*A*). Expression of early viral genes is largely unaffected by PFA, whereas early-late genes are partially inhibited and late genes are completely inhibited (13). Ten-plex tandem mass tag (TMT) technology and MS/MS/MS mass spectrometry of whole-cell lysates enabled precise protein quantification (Fig. 1*A*).

We quantified 8,059 human and 149 viral proteins, and observed good correspondence between proteins modulated during HCMV infection in the absence of PFA and protein expression in our previously published proteomic datasets (10) (*SI Appendix*, Fig. S1). Overall, by 96 h postinfection (hpi), 157 human proteins were down-regulated ≥3-fold in the absence of PFA and “rescued” >2-fold in the presence of PFA (Fig. 1*B* and Dataset S1). Application of DAVID software (14) indicated that these included groups of plasma membrane proteins, proteins with immunoglobulin or cadherin domains, and proteins with functions in viral infection (*SI Appendix*, Fig. S2*A* and Dataset S1). Examples included multiple collagens, ephrins, syndecans, and adhesion molecules, such as junctional adhesion molecule-3 (JAM3), in addition to T cell costimulator CD276 and DNA replication inhibitor and HIV-1 restriction factor SLFN11 (15, 16) (Fig. 1*C*). Additionally, 87 human proteins were both up-regulated ≥3-fold by 96 hpi, yet down-regulated >2-fold in the presence of PFA (*SI Appendix*, Fig. S2 *B* and *C* and Dataset S1), indicating that late-expressed viral proteins can exhibit additional functions in host regulation.

### RL1 Is Necessary and Sufficient for SLFN11 Down-Regulation.

**Fig. 2.**
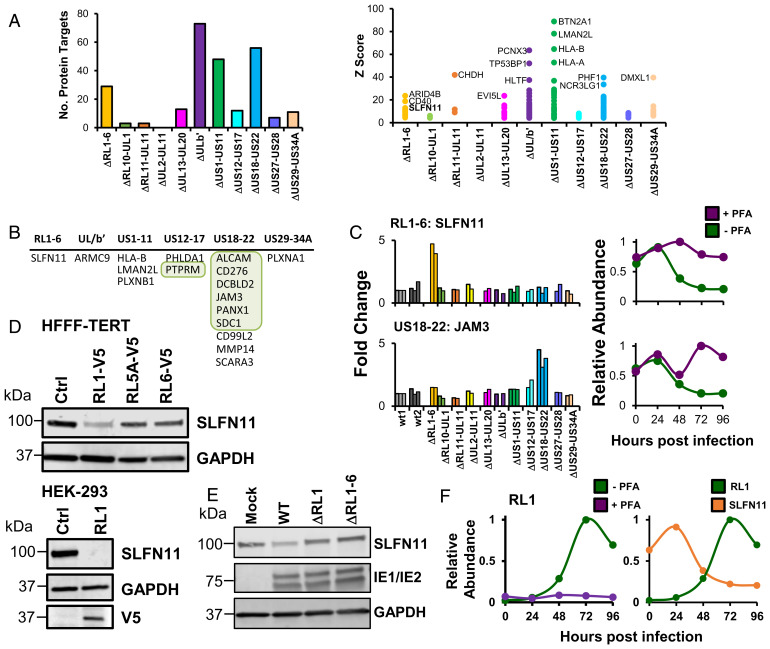
HCMV RL1 is necessary and sufficient for down-regulation of SLFN11. (*A*, *Left*) Numbers of human proteins targeted by each gene block using sensitive scoring (*z*-score > 4 and FC >1.5). For each block, the *z*-scores of all proteins that passed scoring criteria are shown (*Right*). All viruses were examined in duplicate or triplicate across three separate experiments, the first two of which we have published previously (7) (*SI Appendix*, Fig. S3*A*). Infection was at an MOI of 10 for 72 h. Further details are given in *Materials and Methods* and *SI Appendix*, *Materials and Methods*. (*B*) Table of 17 proteins that were down-regulated >3-fold during HCMV infection, rescued >2-fold by PFA (Fig. 1*B*), and passed sensitive scoring criteria to identify the targeting gene block. (*C*) Examples of data for proteins listed in *B*. In the *Left* panels, bars of the same color represent biological replicates (see also *SI Appendix*, Fig. S3*A*). (*D*) Immunoblot confirming that RL1 alone is sufficient for down-regulation of SLFN11 in stably transduced HFFF-TERTs (*Upper*) and transiently transfected HEK-293s (*Lower*). As we reported previously (9), expression of RL5A and RL6 was not detected by immunoblot, whereas both were detected by mass spectrometry (Fig. 3*A* and Dataset S4). (*E*) RL1 is necessary for down-regulation of SLFN11. HFFF-TERTs were infected at an MOI of 10 for 72 h with WT Merlin-strain HCMV, a single ΔRL1 deletion mutant and the ΔRL1-6 block deletion mutant. (*F*) Expression of RL1 during HCMV infection is inhibited by PFA (*Left*). The temporal profile of RL1 expression correlates inversely with expression of SLFN11 during HCMV infection (*Right*). Data for each protein is shown from the PFA screen proteomic experiment (Fig. 1*A*). Although RL1 expression could not be directly validated due to the lack of reagents that detect its expression in the context of HCMV infection, two peptides unique only to RL1 were quantified (*SI Appendix*, Fig. S4).

Identification of which HCMV proteins target a given cellular factor can be challenging due to the substantial coding capacity of HCMV. To identify viral proteins targeting host factors late during HCMV infection, we extended our previous approach that analyzed infection at 72 h with a panel of recombinant viruses, each deleted for one or other of a series of blocks of genes nonessential for replication in vitro (12) (*SI Appendix*, Fig. S3*A*). In this analysis, all viruses were examined in at least biological duplicate, and for the first time ΔRL1-6 HCMV was included since the functions of HCMV factors encoded within this gene block (RL1, RL5A, RL6 proteins, and the RNA2.7 long-noncoding RNA) are poorly characterized. For each human protein, a *z-*score and fold-change (FC) compared to WT infection was calculated (*SI Appendix*, *Materials and Methods*). Sensitive criteria with a final *z*-score of >4 and FC >1.5 assigned 254 modulated cellular proteins to viral blocks (Fig. 2*A*), and stringent criteria (*z*-score > 6, FC > 2) assigned 109 proteins to viral blocks (*SI Appendix*, Fig. S3*B* and Dataset S2). Data from this and the PFA screens are shown in Dataset S3, where the worksheet “Plotter” is interactive, enabling generation of graphs of expression of any of the human and viral proteins quantified.

To identify host factors targeted for down-regulation by late-expressed HCMV proteins, data from the PFA and gene-block screens were combined. Using sensitive criteria, 17 host proteins were down-regulated ≥3-fold by 96 hpi, “rescued” >2-fold by PFA and targeted by one or other of the viral gene blocks examined (Fig. 2*B*). These included proteins with previously described HCMV protein antagonists, for example known targets of the US18-US22 block including ALCAM, CD276, and JAM3, and PTPRM, which is a target of the US12-US17 block (Fig. 2 *B* and *C*) (17). The only assigned target of the RL1-RL6 block that met the threshold for rescue by PFA was SLFN11 (Fig. 2*C*). Furthermore, of the proteins that targeted this block, SLFN11 was the most substantially modulated (*SI Appendix*, Fig. S3*C*).

To determine which viral protein targets SLFN11 for down-regulation, C-terminally V5-tagged RL1, RL5A, and RL6 constructs were stably overexpressed in HFFFs immortalized with human telomerase (HFFF-TERTs). Overexpression of RL1-V5 alone was sufficient for down-regulation of SLFN11 and this was recapitulated by transient transfection of HEK-293 cells with RL1-V5 (Fig. 2*D*). Furthermore, RL1 was necessary for down-regulation of SLFN11 in the context of infection, since neither a single-gene Merlin RL1-deletion mutant nor the RL1–RL6 block deletion recombinant was able to reduce SLFN11 levels (Fig. 2*E*). During HCMV infection, expression of RL1 was completely inhibited by the addition of PFA, and the profile of RL1 expression inversely correlated with the profile of SLFN11 (Figs. 1*C* and 2*F*, and *SI Appendix*, Fig. S4).

### RL1 Degrades SLFN11 Through Recruitment of the Cullin4 E3 Ligase Complex.

**Fig. 3.**
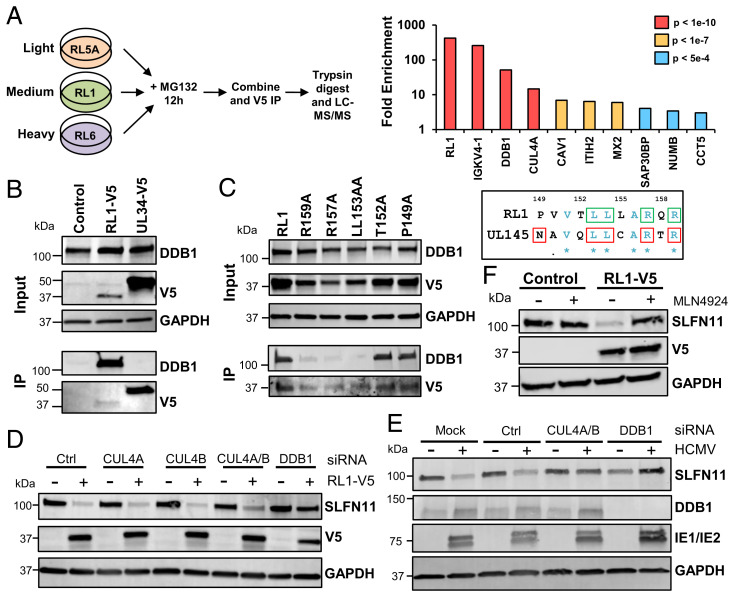
HCMV RL1 degrades SLFN11 via the CRL4 complex. (*A*, *Left*) Schematic of SILAC immunoprecipitation. HFFF-TERTs stably transduced with C-terminally V5-tagged RL1 or RL5A or RL6 as controls were treated with 10 µM MG132 for 12 h prior to harvest. (*Right*) Proteins enriched >3-fold in RL1-expressing cells compared with RL6-expressing cells are shown. *P* values were estimated using significance A values, then corrected for multiple hypothesis testing (38). Full data are shown in Dataset S4. (*B*) Coimmunoprecipitation showing that RL1 interacts with DDB1. HEK-293s were stably transduced with RL1-V5 construct or controls. Input represents 1% of the sample. Proteins were detected with antibodies against V5 and DDB1. (*C*) Coimmunoprecipitation showing that interaction of RL1 and DDB1 is dependent largely on residues conserved between RL1 and UL145 (*Right*) [conserved residues shown in blue; UL145 residues required for interaction with DDB1 in red squares (19); RL1 residues required for interaction with DDB1 in green squares]. HEK-293s were stably transduced with the indicated C-terminally V5-tagged RL1 constructs. Input represents 1% of the sample. Proteins were detected with antibodies against V5 and DDB1. (*D*) Immunoblot showing that SLFN11 down-regulation is dependent on CUL4A, CUL4B, and the adaptor protein DDB1. HFFF-TERTs stably expressing RL1-V5 or control were transfected for 48 h with siRNAs targeted against CUL4A, CUL4B, CUL4A/B, DDB1, or control. (*E*) Immunoblot showing that knockdown of CULA/CUL4B and DDB1 rescues SLFN11 expression during HCMV infection. HFFF-TERTs were transfected for 48 h with siRNA targeted against CUL4A, CUL4B, DDB1, or control and then retransfected for an additional 72 h. (*F*) Inhibition of CRL activity rescues SLFN11 levels. HFFF-TERTs stably transduced with RL1-V5 or control were treated with 1 µM MLN4924 for 24 h prior to harvest.

The HCMV RL1 and UL145 genes are related to each other and thus belong to the RL1 family (6). We and others have previously shown that the UL145 protein can employ CRL4 complex components CUL4A and DDB1 to degrade HLTF and STAT2 (7, 18). Using SILAC (stable isotope labeling with amino acids in cell structure) immunoprecipitation and coimmunoprecipitation, we identified a similar interaction between RL1 and DDB1 and CUL4A (Fig. 3 *A* and *B* and Dataset S4). A panel of alanine substitution mutations was tested to identify the region within RL1 required for interaction with DDB1 based on the DDB1 interaction motif previously identified within UL145 (19) (Fig. 3*C*). As predicted, residues LL153-4, R157, and R159 were required for DDB1 binding, whereas residue T152 was dispensable. In contrast to residue N25 in UL145, which is indispensable for binding DDB1, the equivalent residue P149 in RL1 was not required. This may reflect the differences in the chemical properties of proline and asparagine residues, or the conservation within the DCAF (DDB1-Cullin Accessory Factor) family of asparagine at this position. Residues LL153-4, R157, and R159 are completely conserved across all publicly available HCMV RL1 sequences (263 different strains), and the corresponding residues in HCMV UL145 are also completely conserved (264 different strains). Furthermore, the LLxxRxR motif is highly conserved (complete conservation in seven of eight RL1 orthologs and eight of eight UL145 orthologs) (*SI Appendix*, Fig. S5).

To determine whether the CRL4 complex is required for RL1-mediated degradation of SLFN11, components of the complex were knocked down in HFFF-TERTs stably expressing RL1 or control. Knockdown of DDB1 and CUL4A/4B prevented RL1-mediated loss of SLFN11 (Fig. 3*D* and *SI Appendix*, Fig. S6*A*). These results were recapitulated in the context of HCMV infection (Fig. 3*E* and *SI Appendix*, Fig. S6*B*). SLFN11 was also rescued from degradation in the presence of MLN4924, which prevents the conjugation of NEDD8 on cullins (20), substantiating the requirement for the CRL4 complex in RL1-mediated SLFN11 degradation (Fig. 3*F*). This suggests that RL1 may redirect the Cullin 4 ligase complex to degrade SLFN11, by acting as a viral DCAF.

### SLFN11 Restricts HCMV Infection.

**Fig. 4.**
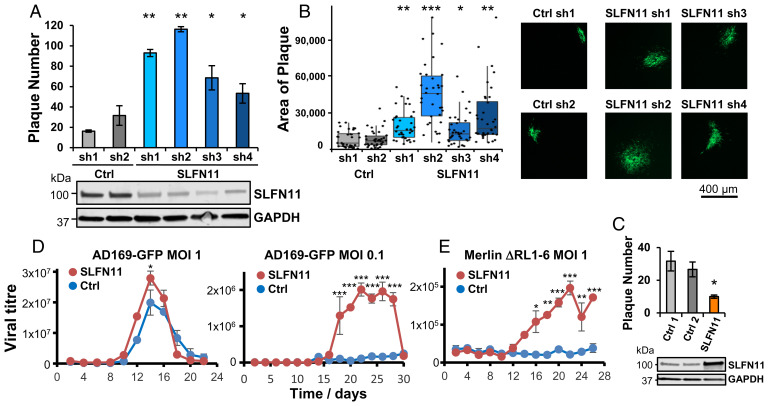
SLFN11 restricts HCMV infection. (*A*) SLFN11 restricts HCMV infection. HFFF-TERTs were stably transduced with shRNAs targeted against SLFN11 or control, and then infected in triplicate with AD169-GFP at an MOI of 0.005 under Avicel for 2 wk before counting the number of plaques. A representative example of two experiments is shown, with error bars showing SD from the mean. *P* values were estimated using a two-tailed *t* test (*n* = 3). *P < 0.05, **P < 0.0005. Immunoblot confirmed knockdown of SLFN11 (*Lower*). (*B*) SLFN11 restricts cell–cell spread of HCMV. Plaque area was calculated using Fiji software (39) using pictures of plaques from the experiment described in *A*. Representative examples are shown (*Right*). *P* values were estimated using a nonparametric Mann–Whitney *U* test (*n* = 30). *P < 0.0005, **P < 0.000005, ***P < 5 × 10^−10^. (*C*) Confirmation that SLFN11 restricts HCMV infection. The experiment was conducted as described in *A*, using HFFF-TERTs stably overexpressing SLFN11 or two independent control cell lines. Immunoblot confirmed overexpression of SLFN11 (*Lower*). *P < 0.05. (*D* and *E*) Multistep growth curves confirm that SLFN11 restricts HCMV infection. HFFF-TERTs stably knocked down for SLFN11 (sh2) or control (ctrl2) were infected in duplicate with AD169-GFP at an MOI of 1 or 0.1 (*D*) or the ΔRL1-6 block deletion mutant at an MOI of 1 (*E*). Culture supernatant was harvested every 2 d and used to infect fresh HFFF-TERTs, where GFP expression at 24 h (AD169-GFP) or 72 h (ΔRL1-6 block deletion mutant) was used to determine viral titer (GFP^+^ cells/mL of supernatant). *P* values were estimated using a paired two-way ANOVA with Tukey’s honestly significant difference test for multiple comparisons (*n* = 2). *P < 0.05, **P < 0.001, ***P < 0.0001.

We sought to determine whether SLFN11 restricts HCMV infection. SLFN11 depletion consistently and significantly increased HCMV replication in four of four independent HFFF-TERT cell lines stably knocked down for SLFN11, in terms of both number and size of plaques (Fig. 4 *A* and *B*). A decrease in the number of plaques was observed upon SLFN11 overexpression (Fig. 4*C*). Multistep growth curves of both RL1-replete and RL1-deficient viruses confirmed a relative replication defect in SLFN11-deficient cells (Fig. 4 *D* and *E*). A greater effect was observed at lower multiplicity of infection (MOI) as we and others have noted during the characterization of other ARFs (7). SLFN11 therefore represents an ARF for HCMV that acts to restrict significantly the spread of HCMV infection.

## Discussion

HCMV and other herpesviruses comprehensively modulate adaptive and innate immunity to facilitate their persistence, employing multiple viral proteins to target cellular factors for degradation (7). Although some viral proteins are expressed throughout the course of infection, others are temporally controlled and target a given host factor at a specific phase of viral replication (7, 11). The present study provides a systematic, searchable database that examines host protein regulation from the point of replication of the viral genome onwards, in addition to identifying which viral gene block targets each of >250 host factors.

The key roles of ARFs in protecting cell populations against HCMV are highlighted by the diversity of proteins with antiviral activity, with different factors affecting distinct steps of the HCMV replication cycle [reviewed in Schilling et al. (21)]. Since description of protein components of promyelocytic leukemia bodies (PML, Sp100, hDaxx) as anti-HCMV ARFs, at least 15 additional ARFs have been identified, including HLTF, Zinc finger antiviral protein (ZAP), the cytidine deaminase APOBEC3A, and the dNTP triphosphohydrolase SAMHD1. Some of these proteins exhibit antiviral activity against diverse viruses, whereas others, such as HLTF, have so far only been associated with restriction of HCMV.

We have now identified SLFN11 as an HCMV restriction factor, although the mechanism of restriction is yet to be determined. SLFN11 inhibits replication of lentiviruses in a codon usage-dependent manner, via its activity as a type II tRNA endonuclease (15, 16, 22, 23). Overall, HCMV genomes exhibit low codon usage bias, although the bias of individual coding sequences varies widely (24). Hu et al. (24) previously determined HCMV codon usage bias on a gene-by-gene basis. However, an analysis of their data using our temporal classification of HCMV protein expression (10) suggested that there is no systematic temporal codon usage bias of HCMV genes. It is possible that RL1-mediated SLFN11 degradation is required for efficient translation of certain poorly codon-optimized late-expressed viral genes. However, expression of poorly codon-optimized early-expressed viral genes would presumably still be reduced irrespective of RL1 expression.

SLFN11 also inhibits translation of certain poorly codon-optimized human genes in the presence of DNA damaging agents, in particular genes specifying the serine/threonine kinases ATM and ATR (25). Both play key roles in the DNA damage response. HCMV requires ATM signaling for efficient replication, although the role of ATR signaling is presently unclear (reviewed in ref. 26). RL1 might thus prevent SLFN11-mediated repression of ATM/ATR in the presence of the DNA damage response stimulated by HCMV infection in order to benefit viral replication. Further alternative mechanisms are suggested by the recent identification of SLFN5 as an ARF for herpes simplex virus 1 (HSV-1) and SLFN14 as an ARF for influenza virus. SLFN5 interacts with HSV-1 viral DNA to repress HSV-1 transcription (27), whereas SLFN14 promotes a delay in viral nucleoprotein translocation from cytoplasm to nucleus and enhances RIG-I mediated interferon-β signaling (28). These observations suggest that other components of the six-member human Schlafen family may act as restriction factors for HCMV, and that Schlafen proteins may more widely restrict other DNA and RNA viruses. Indeed, we found that SLFN5 was down-regulated early during HCMV infection (Dataset S3), raising the intriguing possibility that the virus differentially regulates members of this important family to maximize viral replication.

Several viruses are now recognized to encode factors that degrade host protein targets by subverting cullins or their adaptor proteins, including hepatitis B, HIV, parainfluenza virus, bovine herpesvirus, murine gammaherpesvirus, and CMVs (reviewed in refs. 29 and 30). Including RL1, four CMV proteins have now been recognized to function in this manner, all via recruitment of CRL4 components: murine CMV-encoded M27 and HCMV-encoded UL35 and UL145 (7, 31–33). However, in our recent comprehensive HCMV interactome analysis (9), we detected six additional HCMV proteins that interact with CUL4A or CUL4B (RL12, US7, US34A, UL19, UL122, and UL135), two additional proteins interacting with DDB1 (UL19 and UL27), and three viral proteins interacting with other cullins (US30, UL26, and UL36). These data suggest that there are likely to be additional as yet uncharacterized mechanisms for HCMV-mediated cullin subversion, which may lead to degradation of additional host targets.

The presence of orthologs of RL1 and UL145 in the same positions and orientations in Old and New World monkey and ape cytomegalovirus genomes indicates that this pair of genes has existed for at least 40 million y. Furthermore, the conservation of amino acid residues required for DDB1 interaction suggest that the functions they serve are both ancient and essential for viral replication. Presumably, one or other of these genes developed first (perhaps by a now undetectable gene capture) and then duplicated. Sequences from early primate branches would be required to investigate the evolutionary history further, but these are presently lacking.

Our identification of RL1-mediated SLFN11 degradation provides evidence for direct viral antagonism of this important restriction factor, and might help to explain the evolution of SLFN11 under recurrent positive selection throughout primate development (23). Other mechanisms may also underlie this selection. Schlafen genes acquired by orthopoxviruses might inhibit their host counterparts, possibly by preventing cellular Schlafen multimerization (23, 34). Certain flaviviruses might also encode anti-SLFN11 mechanisms, which could explain the differential susceptibility of West Nile, Zika, and dengue viruses to SLFN11 effects (35). Additionally, sperm–egg interactions and meiotic drive can both result in strong signatures of recurrent positive selection, and some mammalian Schlafen genes have been implicated in sperm–egg incompatability (23, 34).

Only three drugs are commonly used in HCMV treatment, all exhibiting significant adverse effects and the risk of drug resistance. A novel therapeutic approach would be to prevent interaction of virally encoded immune antagonists with their cellular partners. The interaction of RL1 with SLFN11 is one example that could be inhibited for therapeutic effect. Other interactions involving distinct antiviral pathways could be targeted simultaneously to inhibit viral replication potently, for example between HCMV UL145 and HLTF. Alternatively, compounds that inhibit CRL function could be used in anti-HCMV therapy. It has been demonstrated that MLN4924 inhibits HCMV genome replication in vitro at nanomolar concentrations (29), but, to our knowledge, this compound has yet to be tested against HCMV in any clinical setting. Finally, our data are likely to identify further proteins that have roles in restricting infection by HCMV or other viruses.

## Materials and Methods

Extended materials and methods can be found in *SI Appendix*.

### Viral Infections for Proteomic Screens.

HCMV strain Merlin was used in the PFA screen (36). Where indicated, cells were incubated with 300 μg/mL PFA (carrier: water) from the time of infection. For the block deletion mutant screen, 10 of the 11 block HCMV deletion mutants have been described previously (12). The ΔRL1-6 block deletion mutant was generated in the same fashion on the strain Merlin background lacking UL16 and UL18 and expressed a UL32-GFP reporter (wt2) (all viral recombinants used are shown in Dataset S5). Detailed methods for whole-cell lysate protein preparation and digestion, peptide labeling with TMT, HpRP fractionation, liquid chromatography-mass spectrometry, and data analysis are provided in *SI Appendix*.

### Immunoprecipitation.

Cells were harvested in lysis buffer, tumbled on a rotator, and then clarified by centrifugation and filtration. After incubation with immobilized mouse monoclonal anti-V5 agarose resin, samples were washed and then subjected either to immunoblotting or to mass spectrometry (*SI Appendix*).

### Plasmid Construction and Transduction.

Lentiviral expression vectors encoding SLFN11, SLFN11-HA, or the V5-tagged viral proteins RL1, RL5A, RL6, and UL34 (control) were synthesized by PCR amplification and then cloned into Gateway vectors (7). V5-tagged RL1 point mutants were generated by PCR site-directed mutagenesis. For shRNA, two partially complementary oligonucleotides were annealed, and the resulting product was ligated into the pHR-SIREN vector. The primers and templates used are described in Dataset S5. Stable cell lines were generated by transduction with lentiviruses produced via the transfection of HEK293T cells with the lentiviral expression vectors and helper plasmids.

### siRNA Knockdown.

HFFF-TERTs constitutively expressing RL1-V5 or control were transfected with pools of siRNAs for CUL4A, CUL4B, a mixture of CUL4A and CUL4B, DDB1 or nontargeting siRNAs (Dharmafect) with RNAiMAX (Thermo). Cellular lysates were harvested 48 h posttransfection for immunoblotting.

For infection experiments, HFFF-TERTs were transfected twice with pools of siRNA. Forty-eight hours after the first transfection, cells were passaged for retransfection the following day, and cells were infected with WT HCMV 24 h after the second transfection. Cellular lysates were harvested 72 hpi.

### Immunoblotting.

Protein concentration was measured in lysed cells using a bicinchoninic acid (BCA) assay. Aliquots (50 µg) of denatured, reduced protein was separated by SDS/PAGE, transferred to a polyvinylidene difluoride (PVDF) membrane, and probed using the primary and secondary antibodies detailed in *SI Appendix*. Fluorescent signals were detected using the Odyssey CLx Imaging System (LI-COR), and images were processed and quantified using Image Studio Lite V5.2 (LI-COR).

### Plaque Assay.

HFFF-TERTs stably expressing shRNA constructs targeted against SLFN11 or control, or overexpressing SLFN11 or control, were infected in triplicate at an MOI of 0.005 with RCMV-288 (strain AD169 expressing enhanced green fluorescent protein under the control of the HCMV β-2.7 early promoter) (37). The medium was then replaced with a 1:1 (vol/vol) mixture of 2× DMEM and Avicel [2% (wt/vol) in water]. This mixture was removed 2 wk after infection and the cells were washed then fixed in 4% (wt/vol) paraformaldehyde. The number of plaques per well was counted on the basis of GFP fluorescence. Plaque area was calculated using ImageJ Fiji software.

## Supplementary Material

Supplementary File

Supplementary File

Supplementary File

Supplementary File

Supplementary File

Supplementary File

## Data Availability

The mass spectrometry proteomics data have been deposited to the ProteomeXchange Consortium via the PRIDE partner repository with the dataset identifier PXD026785 (40) (http://proteomecentral.proteomexchange.org/cgi/GetDataset?ID=PXD026785). All materials described in this manuscript, and any further details of protocols employed, can be obtained on request from the corresponding author by email to mpw1001@cam.ac.uk.
