# Supplementary material for: Human cytomegalovirus protein RL1 degrades the antiviral factor SLFN11 via recruitment of the CRL4 E3 ubiquitin ligase complex
Source: Proc Natl Acad Sci U S A. 2022 Feb 1;119(6):e2108173119. doi: 10.1073/pnas.2108173119 (PMC8832970; doi:10.1073/pnas.2108173119)
Supplement: Supplementary File [file pnas.2108173119.sapp.pdf]

PNAS
www.pnas.org

Supplementary information for

Human cytomegalovirus protein RL1 degrades the antiviral factor SLFN11 via recruitment of the CRL4 E3 ubiquitin ligase complex

Katie Nightingale[a,b,1], Martin Potts[a,b,1], Leah Hunter[a,b,1], Ceri A. Fielding[c,1], Cassie Zerbe[a,b], Alice Fletcher-Etherington[a,b], Luis Nobre[a,b], Eddie C.Y. Wang[c], Blair L. Strang[d], Jack Houghton[a,b], Robin Antrobus[a,b], Nicolas M. Suarez[e], Jenna Nichols[e], Andrew J. Davison[e], Richard J. Stanton[c], Michael P. Weekes[a,b,2]

Correspondence: Michael P. Weekes

Cambridge Institute for Medical Research, University of Cambridge, Keith Peters Building, Hills Road, Cambridge CB2 0XY, UK

Email: mpw1001@cam.ac.uk

Telephone: +44 1223 767811

This PDF file includes:

Supplementary text (Materials and Methods)
Figures S1 to S6
Legends for Datasets S1 to S5
SI References

Other supplementary materials for this manuscript include the following:

Datasets S1 to S5

Materials and Methods

Cells and cell culture

Primary human fetal foreskin fibroblast cells (HFFFs), HFFFs immortalised with human telomerase (HFFF-TERTs), HEK-293T and HEK-293 cells were grown in Dulbecco's modified Eagle's medium (DMEM) supplemented with 10% (v/v) foetal bovine serum (FBS) and 100 IU/ml penicillin / 0.1 mg/ml streptomycin (DMEM+FBS) at 37°C in 5% CO_2. HFFFs and HFFF-TERTs have been tested at regular intervals since isolation to confirm that human leukocyte antigen (HLA) and MHC Class I Polypeptide-Related Sequence A (MICA) genotypes, cell morphology and antibiotic resistance properties are consistent with the original cells. In addition, growth of the HCMV strain used is limited to a small number of human cell types including fibroblasts, further limiting the chances that the cells have been contaminated with another cell type.

For SILAC immunoprecipitations, cells were grown for seven divisions in DMEM supplemented with 10% (v/v) dialysed FBS, 100 IU/ml penicillin / 0.1 mg/ml streptomycin, 280 mg/l L-proline and either light (Arg 0, Lys 0), medium (Arg 6, Lys 4) or heavy (Arg 10, Lys 8) amino acids at 50 mg/l. Where indicated, cells were treated with 10 mM MG132 (Merck) for 12 h,or 1 µM MLN4924 (Abcam, ab216470) for 24 h prior to harvesting.

Viruses

The parental virus (RCMV1111) used was derived by transfection of a bacterial artificial chromosome (BAC) clone of HCMV strain Merlin, the genome of which is designated the reference HCMV sequence by the National Center for Biotechnology Information and was sequenced after three passages in vitro (1, 2). RCMV1111 contains point mutations in two genes (RL13 and UL128) that enhance replication in fibroblasts (2). Ten of the 11 HCMV block deletion mutants used have been described previously (3) and were generated on a strain Merlin background (wt1) or wt1 that lacked UL16 and UL18 and expressed a UL32-GFP reporter (wt2). The ΔRL1-6 block deletion mutant (RCMV1332) was generated in the same fashion on the wt2 background and validated by whole genome sequencing. The ΔRL1 single gene deletion mutant (RCMV3056) was generated on a wt1 background with a UL36-P2A-GFP reporter. RCMV288 was used for plaque assays, and is based on HCMV strain AD169 with one copy of the EGFP (enhanced green fluorescent protein) gene inserted in one copy of the HCMV long repeat under the control of the HCMV RNA2.7 early promoter (between nucleotides 4576 to 2154 with respect to the strain AD169 genomic sequence) (4). All viral recombinants used are described in **Dataset S5A**.

Virus infections

For the Phosphonoformate (PFA) screen, 1.5 x 10^7 HFFFs were plated in a 150 cm^2 flask 24 h prior to infection with HCMV strain Merlin at MOI 10. Where indicated, cells were incubated with 300 µg/ml PFA from the time of infection. For the block deletion mutant screen, 1 x 10^6 HFFF-TERTs were plated in a 25 cm^2 flask 24 h prior to infection with HCMV strain Merlin at MOI 5 as previously described (3). Mock infections were performed identically but with DMEM instead of viral stock. The zero time point (0 h) was considered to be the time at which cells first came into contact with virus. Cells were incubated with virus for 2 h at 37 °C on a rocking platform, and then the medium was replaced with DMEM+FBS.

Whole cell lysate protein digestion

Cells were washed twice with phosphate buffered saline (PBS), and 250 µl lysis buffer (6 M guanidine, 50 mM HEPES pH 8.5) was added. Cell lifters (Corning) were used to scrape the cells into the lysis buffer, and the suspension was transferred into a 1.5 ml Eppendorf tube, vortexed extensively, and sonicated. Cell debris was removed by centrifuging twice at 21,000 g for 10 min. Half of each sample was kept for subsequent analysis by immunoblot where required. For the other half, dithiothreitol (DTT) was added to a final concentration of 5 mM and samples were incubated for 20 min at room temperature. Cysteine residues were alkylated with 14 mM iodoacetamide and incubated at room temperature for 20 min in the dark. Excess iodoacetamide was quenched with 5mM DTT for 15 mins. Samples were diluted with 200 mM HEPES pH 8.5 to bring the guanidine concentration to1.5 M and digested at room temperature for 3 h with LysC protease at a protease-to-protein ratio of 1:100. Samples were further diluted with 200 mM HEPES pH 8.5 to bring the guanidine concentration to 0.5 M. Trypsin was then added at a protease-to-protein ratio of 1:100, and the reactions were incubated overnight at 37°C. Reactions were quenched with 5% (v/v) formic acid and centrifuged at 21,000 g for 10 min to remove undigested protein. Peptides were subjected to C18 solid-phase extraction (SPE, Sep-Pak, Waters) and vacuum-centrifuged to near-dryness.

Peptide labelling with tandem mass tags (TMTs)

In preparation for TMT labeling, desalted peptides were dissolved in 200 mM HEPES pH 8.5. Peptide concentration was measured by microBCA (Pierce). TMT reagents (0.8 mg) were dissolved in 43 µl anhydrous acetonitrile and 3 µl added to 25 µg of peptide at a final acetonitrile concentration of 30% (v/v). Sample labelling was as indicated in **Dataset S5B**. Following incubation at room temperature for 1 h, the reaction was quenched with hydroxylamine to a final concentration of 0.3% (v/v). The nine TMT-labelled

samples that constituted the PFA screen were combined at a ratio of 1:1:1:1:1:1:1:1:1, and the ten samples that constituted the block deletion mutant screen were combined at a ratio of 1:1:1:1:1:1:1:1:1:1. Combined samples were vacuum-centrifuged to near dryness and subjected to C18 SPE (Sep-Pak, Waters). An unfractionated 'singleshot' sample was analysed initially to ensure similar peptide loading across each TMT channel and avoid the need for excessive electronic normalization. As all normalisation factors were >0.5 and <2, data for the PFA singleshot experiment was analysed with data for the corresponding fractions to increase the overall number of peptides quantified.

Offline high pH reversed phase (HpRp) fractionation and LC-MS/MS/MS for the PFA experiment

Combined, TMT-labeled peptide samples were fractionated using an Agilent 300Extend C18 column (5 µm particles, 4.6 mm ID, 220 mm length) and an Agilent 1100 quaternary pump equipped with a degasser and a photodiode array detector (220 and 280 nm, ThermoFisher, Waltham, MA). Peptides were separated with a linear gradient of 5-35% (v/v) acetonitrile in 10 mM ammonium bicarbonate pH 8 over 60 min. Fractions were recombined orthogonally in a checkerboard fashion, combining alternate wells from each column of the plate into a single fraction, and commencing combination of adjacent fractions in alternating rows. Wells were excluded prior to the start or after the cessation of elution of peptide-rich fractions, as identified from the UV trace. This yielded two sets of 12 combined fractions, A and B. All 12 set A fractions were dried in a vacuum centrifuge, desalted using a StageTip (5) and resuspended in 10 µl MS solvent (4% (v/v) MeCN, 5% (v/v) formic acid) prior to LC-MS/MS/MS.

For LC/MS/MS/MS, peptides were separated on a 75 µm inner diameter microcapillary column packed with 0.5 cm of Magic C4 resin (5 µm, 100 Å, Michrom Bioresources) followed by approximately 20 cm of GP118 resin (1.8 µm, 120 Å, Sepax Technologies). Peptides were separated using a 3 h linear gradient (WCL samples) or 2 h gradient (PM samples) of 6-30% (v/v) acetonitrile in 0.125% (v/v) formic acid at a flow rate of 300 nl/min. Each analysis used an MS3-based TMT method (6, 7). The scan sequence began with an MS1 spectrum (Orbitrap analysis, resolution 120,000, 400−1400 Th, AGC target 2×10^5, maximum injection time 200 ms). 'Top speed' (2s) was selected for MS2 analysis, which consisted of CID (quadrupole ion trap analysis, AGC 4×10^3, NCE 35, maximum injection time 150 ms). The top ten precursors were selected for MS3 analysis, in which precursors were fragmented by HCD prior to Orbitrap analysis (NCE 55, max AGC 5×10^4, maximum injection time 250 ms, isolation specificity 0.5 Th, resolution 60,000) (6).

Offline HpRp fractionation and LC-MS/MS/MS for the block deletion mutant experiment

TMT-labelled tryptic peptides were subjected to HpRP fractionation using an Ultimate 3000 RSLC UHPLC system (Thermo Fisher Scientific) equipped with a 2.1 mm internal diameter (ID) x 25 cm long, 1.7 mm particle Kinetix Evo C18 column (Phenomenex). The mobile phase consisted of A: 3% (v/v) acetonitrile (MeCN), B: 100% MeCN and C: 200 mM ammonium formate pH 10. Isocratic conditions were 90% A/10% C, and C was maintained at 10% throughout the gradient elution. Separations were conducted at 45°C. Samples were loaded at 200 ml/min for 5 min. The flow rate was then increased to 400 ml/min over 5 min, after which the gradient elution proceed as follows: 0-19% B over 10 min, 19-34% B over 14.25 min, 34-50% B over 8.75 min, finally washing with 90% B over 10 min. UV absorbance was monitored at 280 nm, and 15 s fractions were collected into 96 well microplates using the integrated fraction collector. Fractions were recombined orthogonally in a checkerboard fashion (as described above), combining alternate wells from each column of the plate into a single fraction, and commencing combination of adjacent fractions in alternating rows. Wells were excluded prior to the start or after the cessation of elution of peptide-rich fractions, as identified from the UV trace. This yielded two sets of 12 combined fractions, A and B. All 24 fractions were dried in a vacuum centrifuge and resuspended in 10 µl MS solvent (4% MeCN, 5% formic acid) prior to LC-MS/MS/MS.

For LC/MS/MS/MS, the loading solvent was 0.1% (v/v) TFA and the analytical solvents were A: 0.1% (v/v) formic acid (FA) and B: MeCN + 0.1% (v/v) FA. All separations were carried out at 55°C. Samples were loaded at 10 ml/min for 5 min in loading solvent before beginning the analytical gradient. The following gradient was used, whereby A was used to dilute B at the percentage shown: 3-5.6% B over 4 min, 5.6-32% B over 162 min, followed by a 5 min wash with 80% B, a 5 min wash with 90% B and equilibration at 3% B for 5 min. Each analysis used a MultiNotch MS3-based TMT method (McAlister et al., 2014). The following settings were used: MS1: 400-1400 Th, Quadrupole isolation, 120,000 Resolution, $2x10^5$ AGC target, 50 ms maximum injection time, ions injected for all parallisable time. MS2: Quadrupole isolation at an isolation width of m/z 0.7, CID fragmentation (NCE 30) with ion trap scanning out in rapid mode from m/z 120, $1x10^4$ AGC target, 70 ms maximum injection time, ions accumulated for all parallisable time in centroid mode. MS3: in Synchronous Precursor Selection mode the top 10 MS2 ions were selected for HCD fragmentation (NCE 65) and scanned in the Orbitrap at 50,000 resolution with an AGC target of $5x10^4$ and a maximum accumulation time of 150 ms, and ions were not accumulated for all parallelisable time. The entire MS/MS/MS cycle had a target time of 3 s. Dynamic exclusion was set to ±10 ppm for 90 s. MS2 fragmentation was trigged on precursors 5x103 counts and above.

Immunoprecipitation, protein digestion and LC/MS/MS for interaction analysis

Cells were harvested in lysis buffer (50 mM Tris pH 7.5, 300 mM NaCl, 0.5% (v/v) NP40, 1 mM DTT and Roche protease inhibitor cocktail), tumbled for 15 min at 4°C and then centrifuged at 16,100 g for 20 min at 4°C. Lysates were clarified by filtration through a 0.7 µm filter and incubated for 3 h with immobilised mouse monoclonal anti-V5 agarose resin. Samples were washed seven times with lysis buffer and then seven times with PBS pH 7.4. Proteins bound to the anti-V5 resin were eluted twice by adding 200 ml of 250 mg/ml V5 peptide (Alpha Diagnostic International) in PBS at 37°C for 30 min with agitation. Finally, proteins were precipitated with 20% (v/v) TCA, washed once with 10% (v/v) TCA, washed three times with cold acetone and dried to completion using a centrifugal evaporator. Samples were resuspended in digestion buffer (50 mM Tris pH 8.5, 10% (v/v) MeCN, 1 mM DTT, 10 ug/ml trypsin) and incubated overnight at 37°C with agitation. The reaction was quenched with 50% (v/v) FA, subjected to C18 solid-phase extraction, vacuum-centrifuged to complete dryness, and resuspended in 10 µl MS solvent (4% (v/v) MeCN, 5% (v/v) formic acid) prior to LC-MS/MS. For co-immunoprecipitation, the above protocol was followed except that the number of washes was reduced to three with lysis buffer and two with PBS. Proteins bound to the anti-V5 resin were eluted by adding 40 µl of 250 µg/ml V5 peptide in PBS at 37°C for 30 min with agitation. Eluates were separated by SDS-PAGE as described below.

For LC/MS/MS, the loading solvent was 3% MeCN, 0.1% FA and the analytical solvents were A: 0.1% (v/v) FA and B: MeCN + 0.1% (v/v) FA. All separations were carried out at 55°C. Samples were loaded at 5 ml/min for 5 min in loading solvent before beginning the analytical gradient. The following gradient was used: 3-40% B over 29 min followed by a 3 min wash in 95% B and equilibration in 3% B for 10 min. The following settings were used: MS1: 300-1500 Th, 120,000 resolution, $4x10^5$ AGC target, 50 ms maximum injection time. MS2: Quadrupole isolation at an isolation width of m/z 1.6, HCD fragmentation (NCE 35) with fragment ions scanning in the Orbitrap from m/z 110, $5x10^4$ AGC target, 60 ms maximum injection time, ions accumulated for all parallelisable time. Dynamic exclusion was set to ±10 ppm for 60 s. MS2 fragmentation was trigged on precursors $5x10^4$ counts and above.

Plasmid construction

V5-tagged RL1, RL5A, RL6 and UL34 (as a control) were amplified by PCR from adenoviral templates containing the tagged viral gene under the control of the HCMV major immediate early promoter (MIEP). Primers were designed to recognise the 3' end of the MIEP (forward) and the V5 tag (reverse) (**Dataset S5C**). SLFN11 was amplified from HFFF-TERT cDNA using primers designed to recognise the 5' and 3' ends of the gene. For the HA-tagged construct, the reverse primer contained a 6 bp linker followed by the

coding sequence for an HA tag and a stop codon. A control construct was prepared by annealing two oligonucleotides detailed in **Dataset S5C**. Both primers and oligonucleotides had flanking Gateway attB sequences for the purposes of cloning (**Dataset S5C**). PCR employed PfuUltra II Fusion HS DNA polymerase (Agilent). Constructs were subsequently cloned into lentiviral destination vector pHAGE-pSFFV using the Gateway system (Thermo Scientific).

To generate shRNA constructs, two partially complementary oligonucleotides were annealed, with all sequences shown in **Dataset S5C**. The resulting product was ligated as a BamHI–EcoRI fragment into the pHR-SIREN vector (a gift from Prof. Paul Lehner, University of Cambridge) using T4 ligase (Thermo Scientific). All constructed plasmids were transformed into 5-alpha Competent *E. coli* (NEB) and selected on antibiotic-containing luria broth (LB) agar plates. All plasmid inserts were sequenced fully to check for mutations. Two different non-targeting control shRNA sequences are shown in **Dataset S5C**, which also lists the sequences of all primers and oligonucleotides.

Site-directed mutagenesis

A method based on PCR overlap extension was used to generate point mutations in the coding sequence of RL1. Primer sequences spanning the target region were generated that incorporated the desired sequence changes in both forward and reverse orientations. These, along with primers that would anneal at the 5' and 3' ends of the full-length RL1 coding sequence (RL1F and RL1R, respectively) were used to amplify two fragments of RL1, each incorporating the point mutation (**Dataset S5C**). Fragments were purified and assembled into a full-length mutant RL1 coding sequence by a second round of PCR using only RL1F and RL1R. The product was then purified and subcloned as described above.

Stable cell line production

Lentiviral particles were generated by transfection of HEK-293T cells with the lentiviral transfer vector plus four helper plasmids (VSVG, TAT1B, MGPM2, CMV-Rev1B), using TransIT-293 transfection reagent (Mirus) according to the manufacturer's recommendations. Viral supernatant was typically harvested 48 h after transfection, cell debris was removed with a 0.22 µm filter, and target cells were transduced for 48 h and then subjected to antibiotic selection for two weeks.

siRNA knockdown

24 h prior to transfection, 2.5 x 10^5 HFF-TERTs constitutively expressing RL1-V5 or control were plated in 6-well plates. Cells were transfected with a pool of CUL4A siRNAs (L-012610-00, Dharmafect), a pool

of CUL4B siRNAs (L-017965-00, Dharmafect), a pool of DDB1 siRNAs (L-012890-00, Dharmafect), a 1:1 mixture of CUL4A and CUL4B pools, or a pool of non-targeting siRNAs (D-001810-10, Dharmafect) with RNAiMAX (13778030, Thermo), giving a final siRNA concentration of 50 nM. Cellular lysates were harvested for immunoblotting at 48 h post transfection.

For infection experiments, cells were transfected twice with siRNA to ensure efficient knockdown. 0.5 x 10^6 HFFF-TERTs were seeded in T25 flasks and transfected with pools of siRNA, detailed above, at a final concentration of 20 nM. 48 h post transfection, cells were split 1 in 4 for re-transfection the following day. Cells were infected with wt HCMV or mock-infected 24 h after the second transfection. Cellular lysates were harvested 72 h after infection.

Immunoblotting

Cells were lysed with RIPA buffer (Cell Signaling) containing complete protease inhibitor cocktail (Roche), and protein concentrations were measured using a bicinchoninic acid assay (BCA, Pierce). Lysates were reduced with 6 x protein loading dye (375 mM Tris pH 6.8, 12% (w/v) sodium dodecyl sulfate (SDS), 30% (v/v) glycerol, 0.6 M DTT, 0.06% bromophenol blue) for 5 min at 95°C. An amount (50 µg) of protein for each sample was separated by polyacrylamide gel electrophoresis (PAGE) using 4-15% (w/v) TGX precast protein gels (Bio-rad) and then transferred to polyvinylidene fluorid (PVDF) membranes using a trans-blot system (Bio-rad). The following primary antibodies were used: anti-glyceraldehyde 3-phosphate dehydrogenase (GAPDH) (1:10,000, #MAB5718, R&D Systems), anti-V5 (1:2000, #MA5-15253, Thermo), anti-V5 (1:1000, #D3H8Q, CST), anti-SLFN11 (1:500, #HPA023030, Atlas Antibodies), anti-CUL4A (1:1000, #2699S, CST), anti-DDB1 (1:1000, #5428S, CST), anti-CUL4B (1:1000, #ab157103, Abcam) and anti-IE1/2 (1:1000, #ab53495, Abcam). Secondary antibodies were IRDye 680RD goat anti-mouse (925-68070, LI-COR), IRDye 680RD goat anti-rabbit (926-68071, LI-COR), IRDye 800CW goat anti-mouse (926-32210, LI-COR) and IRDye 800CW goat anti-rabbit (925-32211, LI-COR).

Plaque assay

1.3 x 10^5 HFFF-TERTs stably expressing shRNA constructs targeted against SLFN11 or control, or overexpressing SLFN11 or control were plated in 12-well plates in triplicate 24 h prior to infection with AD169-GFP at MOI 0.005. Cells were incubated with virus for 2 h prior to replacing the medium with a 1:1 (v/v) mixture of 2 x DMEM and Avicel (2% w/v in water, FMC BioPolymer). At 2 weeks post infection, the DMEM/Avicel mixture was removed and the cells were washed three times with PBS before fixation

in 4% (w/v) paraformaldehyde. The number of plaques per well was counted on the basis of GFP fluorescence and the average plaque number was calculated across the three wells for each condition.

Plaque size analysis

Images of 12 plaques per well were taken using a Zeiss AxioObserver inverted fluorescence microscope at 5 x magnification. The first 12 plaques encountered that were suitable for imaging were used. Suitable plaques were those that fitted entirely in the field of view, were not located against the edge of the well, and were the only plaque within the field of view. Images were converted into greyscale and plaque area was calculated using Image J Fiji.

Viral growth curves

1.4 x 10^5 HFFF-TERTs stably expressing shRNA constructs targeted against SLFN11 (sh2) or control (Ctrl2) were plated in 12-well plates in duplicate 24 h prior to infection with AD169-GFP at MOI 0.1, or the ΔRL1-6 block deletion mutant at MOI 1. Cells were incubated with virus for 2 h prior to replacing the medium with DMEM-FBS. Culture supernatant was harvested every 2 days, centrifuged at 500g and stored at -80°C. Output virus in the supernatants was titrated as follows: 2 x 10^4 HFFF-TERTs were plated in 96-well plates and infected after 24h with either neat culture supernatants (AD169-GFP MOI 0.1, Merlin ΔRL1-6 MOI 1) or supernatants at a 1:10 dilution in DMEM-FBS (AD169-GFP MOI 1) for 2h prior to replacing the medium with DMEM-FBS. Cells were harvested after 24h (AD169-GFP, since the GFP tag is under the control of the RNA2.7 early promoter) or 72h (Merlin ΔRL1-6, since GFP is tagged to UL32, a late gene). Cells were washed with PBS, trypsinised and fixed with paraformaldehyde. GFP expression was measured using an Accuri C6 flow cytometer (BD) and used to determine viral titre in GFP^+ cells/ml of supernatant.

Data analysis

Mass spectra were processed using a Sequest-based software pipeline for quantitative proteomics, “MassPike”, through a collaborative arrangement with Professor Steven Gygi’s laboratory at Harvard Medical School. MS spectra were converted to mzXML using an extractor built upon Thermo Fisher’s RAW File Reader library (version 4.0.26). In this extractor, the standard mzxml format has been augmented with additional custom fields that are specific to ion trap and Orbitrap mass spectrometry and essential for TMT quantitation. These additional fields include ion injection times for each scan, Fourier transform-derived baseline and noise values calculated for every Orbitrap scan, isolation widths for each scan type,

scan event numbers, and elapsed scan times. This software is a component of the MassPike software platform and is licensed by Harvard Medical School.

A combined database was constructed from (a) the human Uniprot database (26 January 2017), (b) the HCMV strain Merlin Uniprot database, (c) all additional non-canonical human cytomegalovirus ORFs described by Stern-Ginossar et al (8), (d) a six-frame translation of HCMV strain Merlin filtered to include all potential ORFs of ≥8 amino acids (delimited by stop-stop rather than requiring ATG-stop) and (e) common contaminants such as porcine trypsin and endoproteinase LysC. ORFs from the six-frame translation (6FT-ORFs) were named as follows: 6FT_Frame_ORFnumber_length, where Frame is numbered 1-6, and length is the length in amino acids. The combined database was concatenated with a reverse database composed of all protein sequences in reversed order. Searches were performed using a 20 ppm precursor ion tolerance. Fragment ion tolerance was set to 1.0 Th. TMT tags on lysine residues and peptide N termini (229.162932 Da) and carbamidomethylation of cysteine residues (57.02146 Da) were set as static modifications, while oxidation of methionine residues (15.99492 Da) was set as a variable modification. For SILAC analysis, the following variable modifications were used: heavy lysine (8.01420 Da), heavy arginine (10.00827 Da), medium lysine (4.02511 Da), medium arginine (6.02013 Da). SILAC-only searches were performed in the same manner, omitting the TMT static modification.

To control the fraction of erroneous protein identifications, a target-decoy strategy was employed (9). Peptide spectral matches (PSMs) were filtered to an initial peptide-level false discovery rate (FDR) of 1% with subsequent filtering to attain a final protein-level FDR of 1%. PSM filtering was performed using a linear discriminant analysis, as described previously (9). This distinguishes correct from incorrect peptide identifications (IDs) in a manner analogous to the widely used Percolator algorithm (https://noble.gs.washington.edu/proj/percolator/), by employing a distinct machine learning algorithm. The following parameters were considered: XCorr, ΔCn, missed cleavages, peptide length, charge state, and precursor mass accuracy.

Protein assembly was guided by principles of parsimony to produce the smallest set of proteins necessary to account for all observed peptides (algorithm described in (9). Where all PSMs from a given HCMV protein could be explained either by a canonical gene or non-canonical ORF, the canonical gene was picked in preference.

In a few cases, PSMs assigned to a non-canonical gene or 6FT-ORF were a mixture of peptides from the canonical protein and the ORF. This most commonly occurred where the ORF was a 5'-terminal extension of the canonical gene (thus meaning that the ORF was the simplest explanation for the observed peptides).

In these cases, the peptides corresponding to the canonical protein were separated from those unique to the ORF, generating two separate entries. In a single case, PSMs were assigned to the 6FT-ORF 6FT_6_ORF1202_676aa, which is a 5'-terminal extension of the non-canonical ORF ORFL147C. The principles described above were used to separate these two ORFs.

Proteins were quantified by summing TMT reporter ion counts across all matching peptide-spectral matches using "MassPike", as described previously (McAlister et al., 2014). Briefly, a 0.003 Th window around the theoretical m/z of each reporter ion (126, 127n, 127c, 128n, 128c, 129n, 129c, 130n, 130c, 131n) was scanned for ions, and the maximum intensity nearest to the theoretical m/z was used. The primary determinant of quantitation quality is the number of TMT reporter ions detected in each MS3 spectrum, which is directly proportional to the signal-to-noise (S:N) ratio observed for each ion. Conservatively, every individual peptide used for quantitation was required to contribute sufficient TMT reporter ions (minimum of ~500 per spectrum) so that each on its own could be expected to provide a representative picture of relative protein abundance (10). An isolation specificity filter with a cutoff of 50% was additionally employed to minimise peptide co-isolation (10). Peptide-spectral matches with poor quality MS3 spectra (more than 9 TMT channels missing and/or a combined S:N ratio of <100 across all TMT reporter ions) or no MS3 spectra at all were excluded from quantitation. Peptides meeting the stated criteria for reliable quantitation were then summed by parent protein, in effect weighting the contributions of individual peptides to the total protein signal based on their individual TMT reporter ion yields. Protein quantitation values were exported for further analysis in Excel.

For protein quantitation, reverse and contaminant proteins were removed, then each reporter ion channel was summed across all quantified proteins and normalised assuming equal protein loading across all channels. For further analysis and display in Figures, fractional TMT signals were used (i.e. reporting the fraction of maximal signal observed for each protein in each TMT channel, rather than the absolute normalized signal intensity). This effectively corrected for differences in the numbers of peptides observed per protein. For all TMT or SILAC experiments, normalised S:N values are presented in supplementary tables, assuming equal protein loading across all samples. As it was not possible confidently to assign peptides to only two HLA-A, HLA-B or HLA-C alleles, S:N values were further summed to give a single combined result for HLA-A, HLA-B or HLA-C.

Three block viral gene-deletion screens were conducted as described above. For each protein in each screen, a mean (μ) and standard deviation (σ) of all normalised S:N values was calculated. In each case, the maximum (x) value was omitted. For example, for SLFN11 in Figure 2C, μ and σ were calculated using

values for wt1, wt2, RL10-UL1, RL11-UL11, UL2-UL11, UL13-UL20, UL/b', US1-US11, US12-US17, US18-US22, US27-US28, US29-US34A but not the maximum RL1-RL6. The formula $z = (x - \mu) / \sigma$ was then applied to calculate a z-score. Fold change (FC) compared to wild-type (wt) infection was calculated from normalised S:N values using FC = x/wt1. For each experiment, a given protein was initially assigned to the block corresponding to the TMT channel with the maximum S:N. To combine results to assign an overall block to each protein, if the protein was quantified in two or three screens and assigned to the same block, z-scores and fold changes were averaged. If the protein was only quantified in one of the three screens, the block assignment, z-score and fold change from that screen were used. Otherwise, it was not considered possible to assign an overall gene block for that protein.

Hierarchical centroid clustering based on uncentered Pearson correlation, and k-means clustering were performed using Cluster 3.0 (Stanford University) and visualised using Java Treeview (http://jtreeview.sourceforge.net). p-values for protein fold change were estimated using the method of Significance A, calculated in MaxQuant and corrected for multiple hypothesis testing using the method of Benjamini-Hochberg (11). Multiple sequence alignment was performed using Clustal Omega (http://www.ebi.ac.uk/Tools/msa/clustalo/) provided by EMBL-EBI. Cited values for nucleotide and amino acid sequence identity do not include alignment gaps.

Data and materials availability statement

The mass spectrometry proteomics data have been deposited to the ProteomeXchange Consortium (http://www.proteomexchange.org/) via the PRIDE (12) partner repository with the dataset identifier PXD026785. . All materials described in this manuscript, and any further details of protocols employed can be obtained on request from the corresponding author by email to mpw1001@cam.ac.uk.

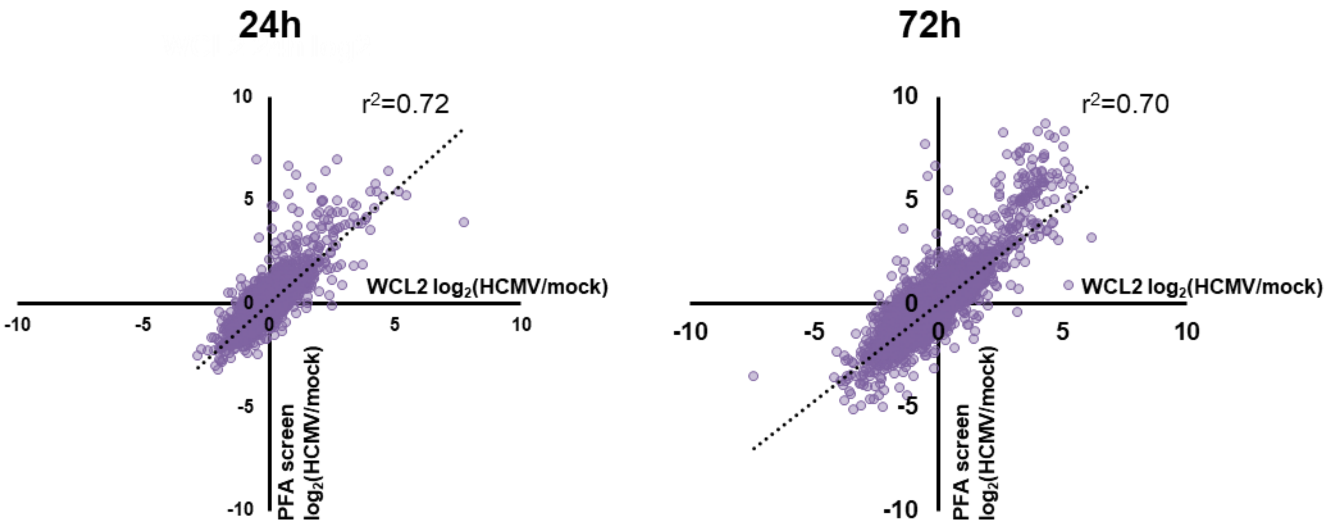

Figure S1

Comparison between data from our previously published temporal analysis of HCMV infection (experiment ‘WCL2’), and the arm of the present experiment (‘PFA screen’) without PFA treatment. Cells were infected with HCMV or mock-infected for 24 or 72 h. HCMV:mock ratios for each protein were highly correlated between experiments.

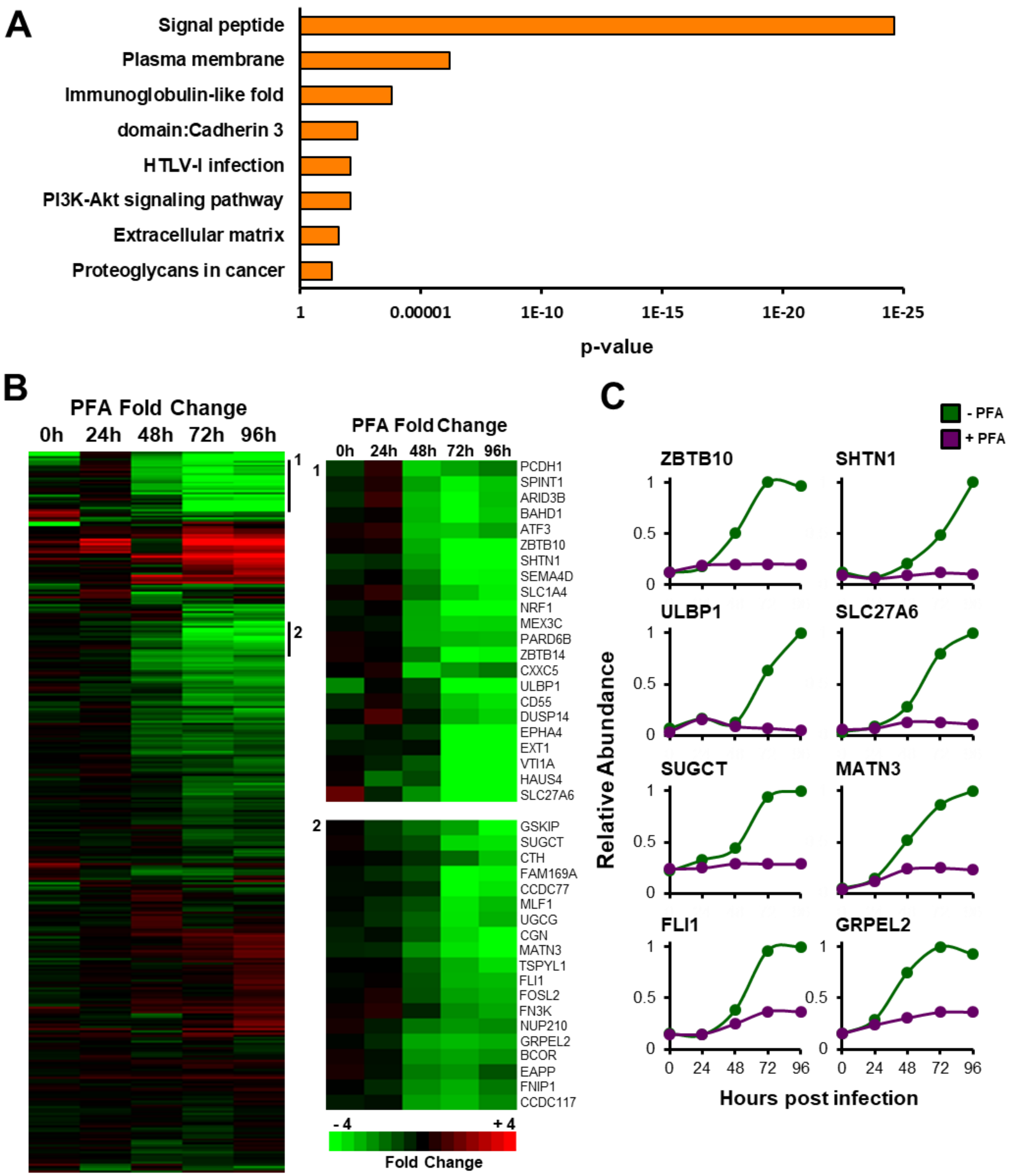
A
Signal peptide
Plasma membrane
Immunoglobulin-like fold
domain:Cadherin 3
HTLV-I infection
PI3K-Akt signaling pathway
Extracellular matrix
Proteoglycans in cancer
1
0.00001
1E-10
1E-15
1E-20
1E-25
p-value
B
PFA Fold Change
0h 24h 48h 72h 96h
1
2
PFA Fold Change
0h 24h 48h 72h 96h
PCDH1
SPINT1
ARID3B
BAHD1
ATF3
ZBTB10
SHTN1
SEMA4D
SLC1A4
NRF1
MEX3C
PARD6B
ZBTB14
CXXC5
ULBP1
CD55
DUSP14
EPHA4
EXT1
VTI1A
HAUS4
SLC27A6
GSKIP
SUGCT
CTH
FAM169A
CCDC77
MLF1
UGCG
CGN
MATN3
TSPYL1
FLI1
FOSL2
FN3K
NUP210
GRPEL2
BCOR
EAPP
FNIP1
CCDC117
- 4
+ 4
Fold Change
C
- PFA
+ PFA
ZBTB10
SHTN1
ULBP1
SLC27A6
SUGCT
MATN3
FLI1
GRPEL2
Relative Abundance
1
0.5
0
0 24 48 72 96
Hours post infection

Figure S2

(A) DAVID analysis of pathway enrichment among 'hits' in the PFA screen (proteins downregulated ≥3-fold by HCMV over the course of infection and rescued >2-fold by the addition of PFA). Benjamini-Hochberg adjusted p-values are shown for each pathway.

(B) Hierarchical cluster analysis of 332 human proteins upregulated ≥3-fold by HCMV over the course of infection. For each protein, the ratios of protein expression in the presence or absence of PFA are shown. Enlargements to the right of the panel show examples of subclusters.

(C) Examples of temporal profiles of proteins whose expression was downregulated in the presence of PFA.

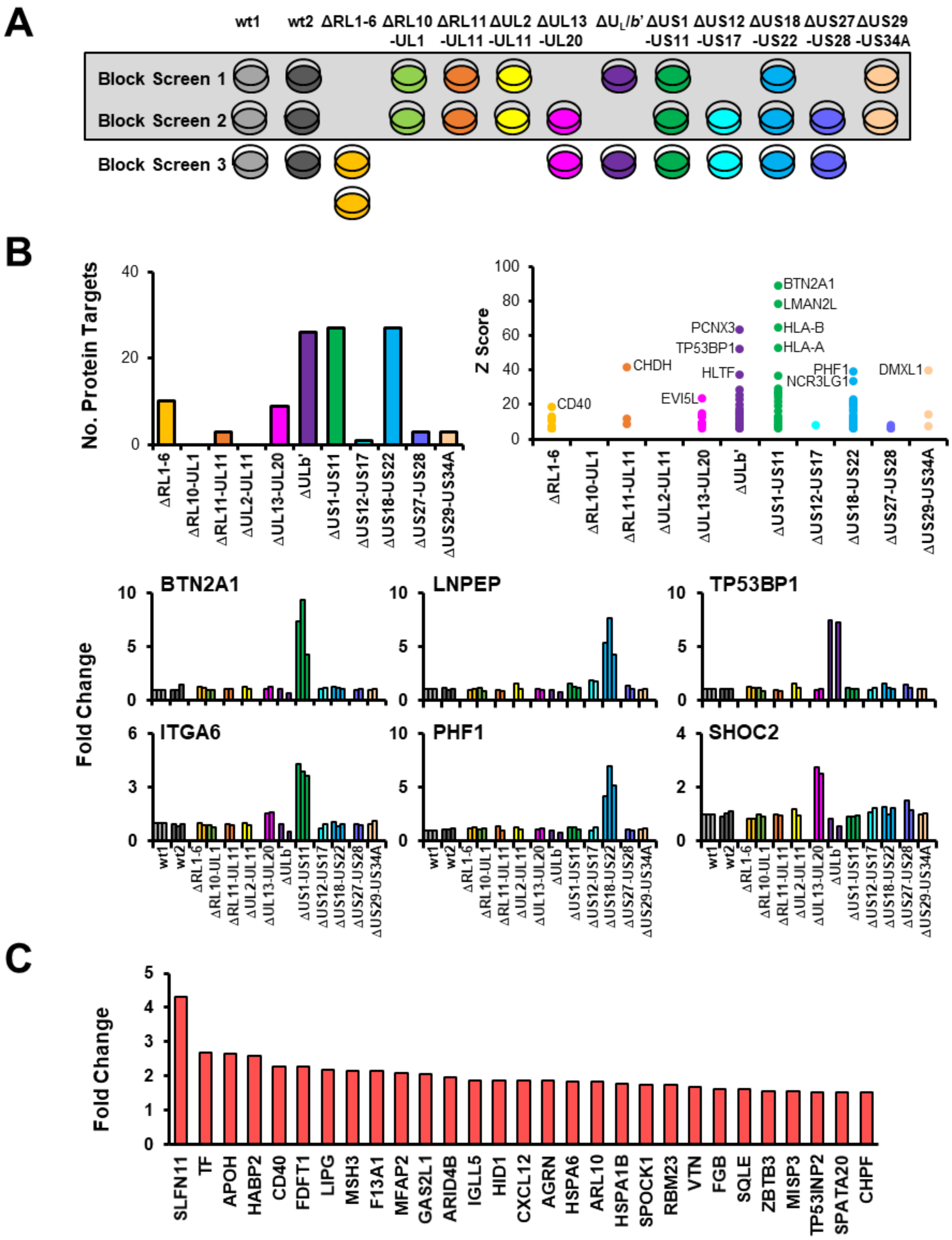
A
wt1
wt2
ΔRL1-6
ΔRL10-UL1
ΔRL11-UL11
ΔUL2-UL11
ΔUL13-UL20
ΔUL/b'
ΔUS1-US11
ΔUS12-US17
ΔUS18-US22
ΔUS27-US28
ΔUS29-US34A
Block Screen 1
Block Screen 2
Block Screen 3
B
No. Protein Targets
Z Score
BTN2A1
LMAN2L
HLA-B
HLA-A
PCNX3
TP53BP1
HLTF
CHDH
CD40
EVI5L
PHF1
NCR3LG1
DMXL1
Fold Change
BTN2A1
LNPEP
TP53BP1
ITGA6
PHF1
SHOC2
C
Fold Change
SLFN11
TF
APOH
HABP2
CD40
FDFT1
LIPG
MSH3
F13A1
MFAP2
GAS2L1
ARID4B
IGLL5
HID1
CXCL12
AGRN
HSPA6
ARL10
HSPA1B
SPOCK1
RBM23
VTN
FGB
SQLE
ZBTB3
MISP3
TP53INP2
SPATA20
CHPF

Figure S3

(A) Schematic of the gene block deletion experiment. Experiments 1 and 2 were reported previously (7), and experiment 3 was performed for the present study, for the first time including the ΔRL1-RL6 gene block deletion mutant, in biological duplicate.

(B) (top left panel) Numbers of human proteins targeted by each block using stringent scoring (z-score >6 and fold change >2). For each block, the z-scores of all proteins that passed scoring criteria are shown (top right panel). Further details of the results using stringent scoring are shown in **Figure 2A** and in the Materials and Methods. (bottom panels) Further examples of results (see also **Figure 2C**). Bars of the same colour represent biological replicates (Figure S3A).

(C) Bar chart illustrating proteins 'rescued' upon deletion of the RL1-RL6 gene block. Fold change values shown were calculated by (average signal : noise (RL1-RL6 gene block deletion mutant) / signal : noise (parental HCMV)). Each protein with a fold change of >2 is illustrated.

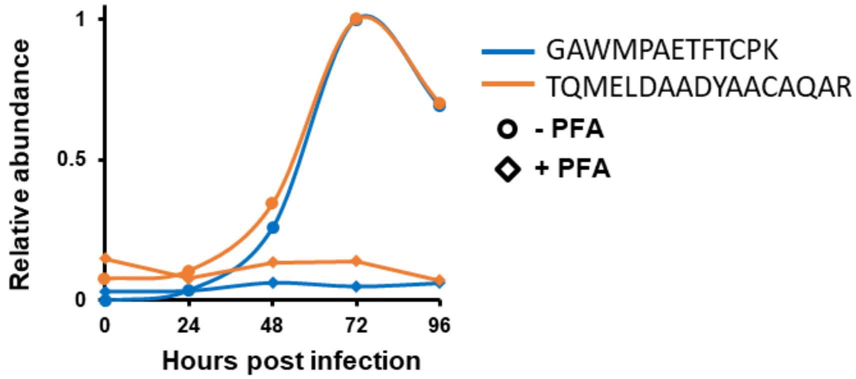

Figure S4: Peptide data for Figure 2E. Two unique RL1 peptides were quantified in the 'PFA screen' proteomic experiment.

A

```
AoBHV1     ~~~ATCWCLWLSRREG---TSARAPRSGSAVHLLAARTRDGH---ADWAFMRARALCL~~~RYRLALLLGGHGQRLQLERATLDEAEERGWLPNIAGRD~~~YERRQGTWMPSETFPTPLL~~~
SaBHV4     ~~~SSCWCIWTPRWPLPGRVTRKRREERGAVHLLNSRARDGR---ADWTFLRVRSLVL~~~RYRIALLFGGHGAHLMIIRPTFDEAASRGLFPRMACQD~~~LCIRRGTWIASETFPTPLL~~~
HuBHV5     ~~~ADCWCMWGRFGTM---------GRQPVVTLLLARQRDGL---ADWNVVRCRGTGF~~~RQHLVFLLGGHGRRVQLERPSAGEAQARGLLPRIRITP~~~RRGERGAWMPAETFTCPKD~~~
PnBHV2     ~~~EDCWCMWGRFGTL---------NRRPVVRLLLQRQLEGR---CDWNVVRCRGMGF~~~RQHLVFLLGGHGQRVQLDRPSAGEAEARGLLPRFHLKP~~~RRGERGAWMPAETFTCPQG~~~
McBHV3     ~~~PNCWCLWGVHAHK---------NHRPIIPLLTARARDGPIKSGGWDVLYGRGYGY~~~TQHIVFLLGGHGPRLQLNRACAREAEARGLLPLWPVHS~~~ERAERGAWIVSDTFTAPGL~~~
McBHV8     ~~~PNCWCLWGVHAHK---------NHRPIISLLTARARDGPIKSGGWDVLYGRGYGY~~~TQHIVFLLGGHGPRLQLNRACAREAEARGLLPFWPVPS~~~ERAERGAWIVSDTFTAPGL~~~
CeBHV5     ~~~PDCWCLWGEHAQK---------AARPLVPLLISRARDGPEKAGAWDQLHGRGFGY~~~TQHVVFLLGGHGARLQLNRPSAREAEARGLLPRWPMHP~~~ERGERGAWIVADTFTAPGN~~~
MdBHV1     ~~~PDCWCLWGCEAEK---------AQRPIVPLLVSRARDGPIKSGGWDVLRGRGYGY~~~TQHVVFLLGGHGPRVHLNRPSAREAEARGLLPRWPMRP~~~ERGERGAWIVSDTFTAPGR~~~
PaBHV4     ~~~PDCWCLWGEYAEK---------AQRPIVPLLVSRTRDGPMKSGAWDVLRGRGYGY~~~TQHVVFLLGGHGPRVHLNRPSAREAEARGLLPRWPMRP~~~ERGERGAWIVTDTFTAPGR~~~
                                           **  * *
```

B

```
AoBHV1                         MPSRNFPQNTTHLLCQRPRDGP---GRWERLIDHVSLIKNYTIATTLIAGTNSCYILLEREGAFAAFRQGLIPEKVRARLKHSEWIQGAWIVSETFPSENVKIYTPPKIYMPS
SaBHV4                       MTTRRRLLPHNTTDLLCKRPRDGP---GRWERLIDHVSLIQDSSLSATLVAGSDNCFILLEREGAFAAFHKGLIPEKTRAKLKGSEWIPGAWIVAETFPDDRVYICTFPETSSCSTSSEIHRE
HuBHV5         MYGVLAHYYSFISSPSVMVNFKHHNAVQLLCARTRDGT---AGWERLTHHASYHANYGAYAVLMATSQRKSLVLHRYSAVTAVALQLMPVEMLRRLDQSDWVRGAWIVSETFPTSDPKGFWSDDDSPMGGSED
PnBHV2     MHDQHAVSAAHYYSFISGPSAMVHYRHQNAVQLLCARARDGT---SGWERLTHHAAYQADYGAYAVLMATSQTKSLVLHRYSAVTAVALQLMPSNVLARLDQSDWVKGAWIVSETFPTCDPDGFRDRRSSSSGDEGENDEGDKRHEGREGESAVGGGDA
McBHV3                          MVFYQYHNAVQLLSARSRDGP---GRWERLTHHASCADDHGACTVLMATSQKRSLVLRRHSAITAVTLRLIPSKILKKLEESDWIPGAWIASETFPTSDAVSGL
McBHV8                          MVFYQYHNAVQLLSARSRDGP---GRWERLTHHASCADDHGACTVLMATSQKRSLVLRRHSAITAVTLRLIPSKILKKLEESDWIPGAWIASETFPTSDVVSGL
CeBHV5                          MVFYQYRNAVQLLSARPRDGP---GRWERLTHHAAYAADHGACTVLMATSQRLSLVLRRHSAISAVTLRLIPAKVLKKLEESEWIPGAWIASETFPTSDVI
MdBHV1                          MVLYQYRNAVQLLSARPRDGP---GRWERLTHHAAYTADHGACTVLMATSQRRSLVLRRHSAISAVTLRLIPDKILKKLEVSDWVPGAWIASETFPTSDISGLR
PaBHV4                          MVFIQYHNAVQLLSARPRDGP---GRWEHLTHHASYTADHGACTVLMATSRKRSLVLRRHSAISAVTLRLIPDKILKKLEVSDWVPGAWIASETFPTSDVSP
CeBHV5                            EEE   HHHHHH       ---  HHHHHHHHHHHH   EEEEEE     EEEEEEE   HHHHHH   HHHHHHHH         EEE
```

C

New World primate viruses

AoBHV1	aotine betaherpesvirus 1	owl monkey	FJ483970
SaBHV4	saimiriine betaherpesvirus 4	squirrel monkey	FJ483967

Old World primate viruses

HuBHV5	human betaherpesvirus 5	human	AY446894
PnBHV2	panine betaherpesvirus 2	chimpanzee	AF480884
McBHV3	macacine betaherpesvirus 3	rhesus macaque	AY186194
McBHV8	macacine betaherpesvirus 8	cynomolgus macaque	JN227533
CeBHV5	cercopithecine betaherpesvirus 5	green monkey	FJ483969
MdBHV1	mandrilline betaherpesvirus 1	drill monkey	KR297253
PaBHV4	papiine betaherpesvirus 4	olive baboon	KR351281

Figure S5. RL1 and UL145 amino acid sequence alignments

(A) RL1 alignment. Omitted subsequences of various lengths are indicated by ~~~ shaded in cyan, and gap characters are indicated by -. Residues fully conserved in the RL1 and UL145 proteins are shaded green, residues fully conserved in one of these proteins and partially in the other are shaded grey, and residues fully conserved in one of these proteins but not the other are shaded yellow. Conserved residues in the LLxxRxR motif are marked by red asterisks.

(B) UL145 alignment. Complete sequences are shown. In addition to the formatting described in (A), possible alternative initiating M residues are in bold font, and the final line in red font shows a prediction of the secondary structure of one sequence computed using Jnet implemented in Jpred 4 (https://www.compbio.dundee.ac.uk/jpred/; H, α-helix; E, extended β-sheet).

(C) List of the abbreviations of virus names, the hosts of the viruses and the GenBank accession nos. of source DNA sequence data.

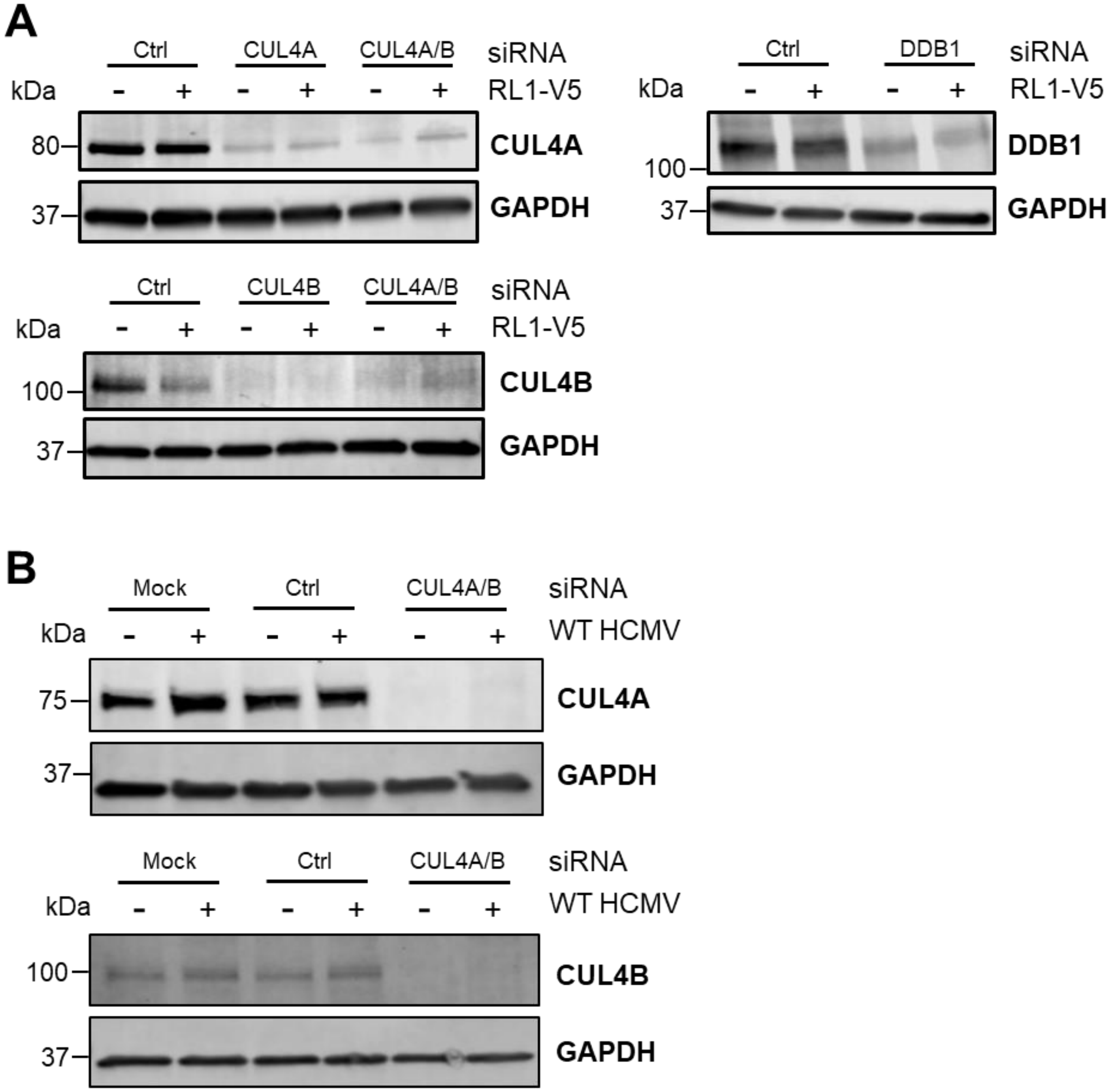

Figure S6.

(A) Demonstration of siRNA-mediated knockdown of CUL4A, CUL4B and DDB1, for the experiment shown in **Figure 3D**.

(B) Demonstration of siRNA-mediated knockdown of CUL4A and CUL4B, for the experiment shown in **Figure 3E**.

Dataset S1 (separate xls. file).

A. Proteins identified by the PFA screen as downregulated >3-fold by 96 hpi and 'rescued' >2-fold by PFA, with both fold changes significant at $p<0.1$ (see Figure 1B).

B. Enrichment of functional pathways within proteins identified in (A), compared to all proteins quantified from this screen.

C. Proteins identified by the PFA screen as upregulated >3-fold by 96 hpi yet inhibited >2-fold by PFA, with both fold changes significant at $p<0.1$ (see Figure 1B).

Dataset S2 (separate xls.file). Predicted block of viral genes targeting 254 human proteins.

Dataset S3 (separate xls. file). Interactive spreadsheet of all data in the manuscript. The "Plotter" worksheet generates graphs for all the human and viral proteins quantified, and visualises statistics. The "Data" worksheet shows minimally annotated protein data, for which the only modifications are formatting, normalization, reassignment of non-canonical HCMV ORFs and combination of peptide data for HLA- A, -B and -C alleles into single combined results, as described in Materials and Methods. The "Lookup" worksheet is used to generate graphs shown in "Plotter".

Dataset S4 (separate xls. file). Full results of the SILAC immunoprecipitation of RL1, RL5A and RL6 (see also **Figure 3A**).

Dataset S5 (separate xls. file).

A. Details of viruses and viral block deletion recombinants used in this study.

B. Details of TMT labelling for each proteomic experiment.

C. Details of oligonucleotides for gene knock-down, overexpression or mutagenesis.

SI References

1. Dolan A, *et al.* (2004) Genetic content of wild-type human cytomegalovirus. *Journal of General Virology* 85(Pt 5):1301-1312.
2. Stanton RJ, *et al.* (2010) Reconstruction of the complete human cytomegalovirus genome in a BAC reveals RL13 to be a potent inhibitor of replication. *Journal of Clinical Investigation* 120(9):3191-3208.
3. Nightingale K, *et al.* (2018) High-definition analysis of host protein stability during Human Cytomegalovirus infection reveals antiviral factors and viral evasion mechanisms. *Cell Host Microbe* 12:447-460.
4. McSharry BP, Jones CJ, Skinner JW, Kipling D, & Wilkinson GW (2001) Human telomerase reverse transcriptase-immortalized MRC-5 and HCA2 human fibroblasts are fully permissive for human cytomegalovirus. *J Gen Virol* 82(Pt 4):855-863.
5. Rappsilber J, Mann M, & Ishihama Y (2007) Protocol for micro-purification, enrichment, pre-fractionation and storage of peptides for proteomics using StageTips. *Nature protocols* 2(8):1896-1906.
6. McAlister GC, *et al.* (2012) Increasing the multiplexing capacity of TMTs using reporter ion isotopologues with isobaric masses. *Analytical Chemistry* 84(17):7469-7478.
7. Ting L, Rad R, Gygi SP, & Haas W (2011) MS3 eliminates ratio distortion in isobaric multiplexed quantitative proteomics. *Nat Methods* 8(11):937-940.
8. Stern-Ginossar N, *et al.* (2012) Decoding human cytomegalovirus. *Science* 338(6110):1088-1093.
9. Huttlin EL, *et al.* (2010) A tissue-specific atlas of mouse protein phosphorylation and expression. *Cell* 143(7):1174-1189.
10. McAlister GC, *et al.* (2014) MultiNotch MS3 enables accurate, sensitive, and multiplexed detection of differential expression across cancer cell line proteomes. *Anal Chem* 86(14):7150-7158.
11. Cox J & Mann M (2008) MaxQuant enables high peptide identification rates, individualized ppb-range mass accuracies and proteome-wide protein quantification. *Nature Biotechnology* 26(12):1367-1372.
12. Vizcaino JA, *et al.* (2016) 2016 update of the PRIDE database and its related tools. *Nucleic acids research* 44(D1):D447-456.
